# Protective human antibodies against Powassan virus

**DOI:** 10.1128/jvi.02105-25

**Published:** 2026-04-30

**Authors:** Georgia Fallon, Stefanie P. Muraro, Samuel Ailsworth, Sean Hui, Ryan J. Malonis, Alexandra L. Tse, Emily Happy Miller, Kartik Chandran, Daved H. Fremont, Michael S. Diamond, Jonathan R. Lai

**Affiliations:** 1 Department of Biochemistry, Albert Einstein College of Medicine 548274, Bronx, New York, USA; 2 Department of Medicine, Washington University School of Medicine 12275, St. Louis, Missouri, USA; 3 Department of Pathology and Immunology, Washington University School of Medicine 12275, St. Louis, Missouri, USA; 4 Department of Microbiology and Immunology, Albert Einstein College of Medicine, Bronx, New York, USA; 5 Department of Medicine, Albert Einstein College of Medicine, Bronx, New York, USA; 6 Department of Molecular Microbiology, Washington University School of Medicine 12275, St. Louis, Missouri, USA; 7 Department of Biochemistry and Molecular Biophysics, Washington University School of Medicine, St. Louis, Missouri, USA; University of Michigan Medical School, Ann Arbor, Michigan, USA

**Keywords:** human antibodies, monoclonal antibody, orthoflavivirus, Powassan virus

## Abstract

Powassan virus (POWV) is a tick-borne orthoflavivirus that can cause severe neuroinvasive disease. There are no approved vaccines or therapeutics, and the incidence of POWV infection in humans is rising. Here, we isolated and characterized a panel of human monoclonal antibodies (mAbs) from a convalescent donor using single B-cell sorting with POWV EDIII and full-length E ectodomain as antigen bait. Binding and neutralization assays with both POWV reporter virus particles and an authentic strain identified multiple neutralizing mAbs that target distinct epitopes across the E glycoprotein, including EDIII lateral ridge/C-C' loop and non-EDIII regions. Four neutralizing mAbs were evaluated in a lethal mouse challenge, two of which conferred substantial protection. These findings define key targets of the human antibody response to POWV and highlight candidates for the development of human monoclonal antibody therapy for this emerging virus.

**IMPORTANCE:**

Powassan virus is an emerging tick-borne orthoflavivirus with steadily increasing case numbers. With an approximately 10% case-fatality rate and no approved therapeutics or vaccines available, POWV represents a potential public health threat. Our results showing that human monoclonal antibodies can protect mice against POWV in a lethal challenge model provide a foundation for developing future effective immunotherapies against this virus.

## INTRODUCTION

Powassan virus (POWV) is a tick-borne orthoflavivirus (TBFV) in the family *Flaviviridae* of enveloped positive-strand RNA viruses (1). First reported in Canada in 1958, POWV is an emerging virus, and the tick vectors that carry it are expanding in range in North America and Russia (2). In humans, POWV infection can cause neuroinvasive disease, including encephalitis, meningitis, and meningoencephalitis (3). The case-fatality rate of POWV infection is 10%, with approximately 50% of survivors suffering long-term neurological damage (3). Both the number and geographic breadth of POWV cases have increased in recent decades. From the discovery of POWV in 1958 until 1998, there were 27 reported POWV cases in both the United States and Canada (4). However, in 2024 alone, there were 54 reported POWV cases in the United States (5). There is no standard diagnostic protocol, and public awareness of POWV is limited. Thus, POWV cases are likely highly underreported. The increasing incidence of POWV infection highlights a need for the development of therapeutics or vaccines, which currently are lacking.

There are two serologically and clinically indistinguishable genetic lineages of POWV, POWVI and POWVII (6). While the two lineages share 96% amino acid identity in their envelope (E) proteins, they are transmitted via different tick families (6). The predominant vector for POWVI is *Ixodes cookei*, whereas POWVII is primarily transmitted by *Ixodes scapularis* deer ticks (7). *I. scapularis* ticks are more aggressive in biting larger mammals, such as humans, and thus POWVII has a greater risk of spreading in endemic regions. In the United States, these tick species are mainly found in the Northeast; however, they are also present in central and southern states (8, 9). Environmental changes, such as increases in temperature and longer warm seasons, could also contribute to an increase in the geographical range of these ticks (9).

**Fig 1:**
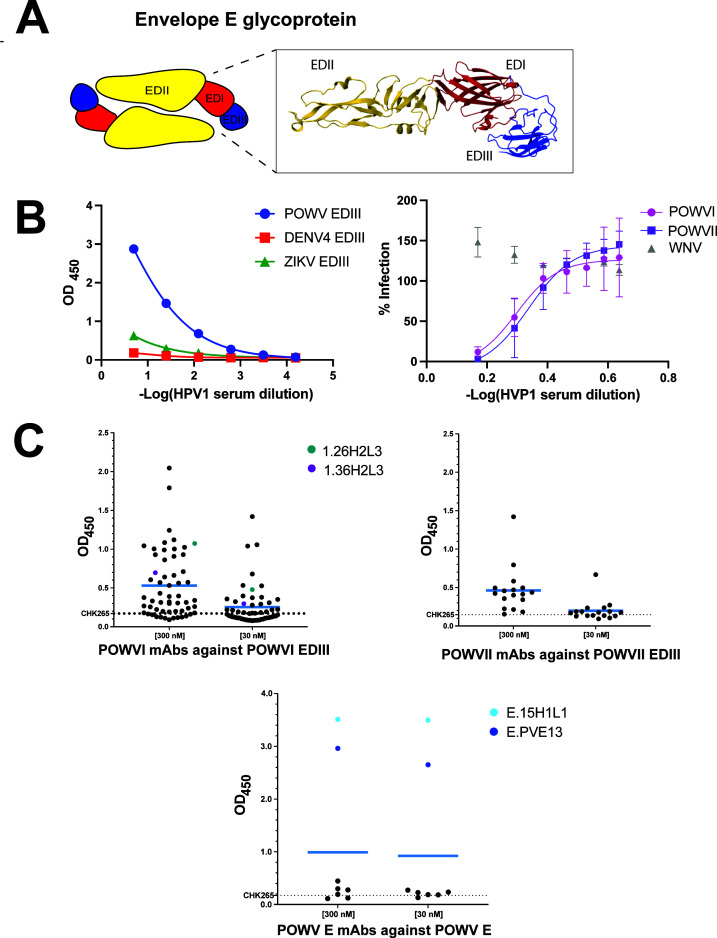
POWV E glycoprotein architecture and overview of HVP1 sera and human mAbs. (**A**) Schematic of POWV E glycoprotein homodimer, which consists of two copies each of the three domains (EDI, EDII, and EDIII). The inset shows the structure of tick-borne encephalitis virus E glycoprotein with the three E domains (PDB: 1SVB). (**B**) Reactivity of HVP1 sera from convalescent patients toward POWVI EDIII in comparison to DENV4 EDIII and ZIKV EDIII. Inhibition of infection POWVI and POWVII reporter virus particles (RVPs) by HVP1 sera in comparison to WNV RVP. (**C**) Plots of ELISA (OD_450_) for the isolated POWVI EDIII, POWVII EDII, and POWV E-sorted mAbs at 300 nM and 30 nM against their respective sorting antigens. The mAbs tested *in vivo* (1.26H2L3, 1.36H2L3, E.15H1L1, and E.PVE13) are indicated. Binding of the negative control CHK265 is indicated by the dashed line. Each data point represents the mean from two or more independent experiments done in triplicate.

The orthoflavivirus virion is a ~50 nm particle composed of three structural proteins (capsid [C], precursor membrane [prM], and E glycoprotein), the viral genomic RNA, and a host lipid envelope (10). The E glycoprotein mediates viral entry and membrane fusion (11). On mature virions, 90 antiparallel E protein homodimers, each 170 Å in length, are organized in icosahedral symmetry axes across the viral membrane (11). The ectodomain of each E monomer is comprised of three domains: EDI, EDII, and EDIII (Fig. 1A). The centrally located EDI, which forms an eight-stranded β-barrel structure, acts as a link between EDII and EDIII (11, 12). EDII contains the highly conserved fusion loop that is critical for membrane fusion with the host cell. The EDIII domain adopts an immunoglobulin-like fold and undergoes substantial conformational change during fusion and is implicated in host cell receptor binding for many orthoflaviviruses (11–18). Although neutralizing antibodies can bind various epitopes on the orthoflavivirus E glycoprotein, EDIII has been shown to be the target of neutralizing antibodies for several different orthoflaviviruses, including POWV (11, 14, 17–20).

Although TicoVac is a licensed vaccine for tick-borne encephalitis virus (TBEV), only supportive care is available for those infected with all other TBFVs, including POWV. Additional vaccines have been developed against mosquito-borne orthoflaviviruses, including yellow fever (YFV), dengue (DENV), and Japanese encephalitis (JEV) viruses (21). Several vaccine platforms have been investigated for POWV, including lipid-encapsulated mRNA and plasmid DNA vectors, virus-like particles (VLPs) presenting the POWV prM and E glycoproteins, attenuated viral strains, and chimeric yellow fever virus-based vaccine constructs (7, 22–25, 26). These vaccine candidates elicit neutralizing and protective antibody responses in mice. However, the prM and E proteins contain many antigenic sites, some of which are targeted by antibodies that are poorly reactive and/or poorly neutralizing. Experience with other orthoflaviviruses demonstrates that conserved sites in EDII and the prM are the targets of broadly cross-reactive yet poorly neutralizing antibodies that might contribute to antibody-dependent enhancement (ADE) (27–29). EDIII-based subunit vaccine approaches have mitigated the risk of inducing non-neutralizing and/or ADE-prone antibodies for mosquito-borne flaviviruses, such as DENV (30–32) and Zika virus (ZIKV) (33–35). Indeed, our laboratory has also previously reported a protective, self-assembling nanoparticle vaccine containing POWV EDIII (36).

Neutralizing mAbs have the potential to protect directly against POWV infection or inform vaccine design through reverse vaccinology approaches. In mice vaccinated with an mRNA encoding POWV prM/E, a panel of neutralizing EDIII-specific mAbs was isolated and used to define key protective sites on both EDII and EDIII (7, 18, 37). Similarly, upon immunization of mice with a POWV EDIII-based nanoparticle, we previously isolated four neutralizing mouse mAbs that bind to the lateral ridge/C-C' loop epitope on EDIII (36).

Here, we describe the isolation and characterization of a panel of human POWV mAbs. Several of these mAbs neutralized infection *in vitro* and bound to different epitopes, with three binding in EDIII and one targeting elsewhere on the full E glycoprotein. Four of the human mAbs were tested in a lethal challenge mouse model with POWVII strain MA51240. We found that two of the human mAbs (one targeting EDIII and the other EDI/II) conferred high levels of protection against POWV. These results help to define the human antibody response to POWV infection, as well as provide human mAbs for potential immunotherapeutic use.

## RESULTS

### Single B-cell cloning and screening of POWV human mAbs

We obtained samples from a seropositive donor who contracted POWV while hiking in the New York Hudson Valley (patient HVP1, Hudson Valley, Powassan). HVP1’s syndrome began as fatigue, fever, and headache, but progressed to severe meningitis. Serum samples from HVP1 obtained 2–5 years post-infection demonstrated strong reactivity to recombinant POWVI EDIII with no reactivity to DENV or ZIKV EDIII, indicating the existence of circulating POWV-specific antibodies (Fig. 1B). Background signal was determined using wells incubated without antigen and with 1% bovine serum albumin (BSA) as a negative control. Reactivity was interpreted based on the relative magnitude and dilution-dependent behavior of ELISA signals across antigens rather than a predefined optical density cutoff. Serum was also tested for neutralizing activity against POWVI and POWVII reporter virus particles (RVPs). These pseudo-infectious viruses are competent for a single round of infection and express GFP, allowing their use under BSL2 conditions. The HVP1 sera neutralized infection of both POWVI and POWVII RVPs but lacked activity against RVPs displaying WNV structural glycoproteins. These results establish the presence of POWV-specific, neutralizing antibodies in the patient serum.

Using fluorescently activated cell sorting (FACS), peripheral blood mononuclear cells (PBMCs) from HVP1 were sorted for POWVI and POWVII EDIII-reactive B cells. Biotinylated EDIII complexed with PE-streptavidin was chosen as the sorting “bait” antigen, as potently neutralizing antibodies against mosquito-borne orthoflaviviruses, such as DENV, ZIKV, and WNV, can target EDIII (17, 38–40). As HVP1’s serum antibodies reacted to both POWVI and POWVII EDIII, we hypothesized that use of POWV EDIII as the sorting antigen could facilitate the isolation of neutralizing antibodies. PBMCs were first gated based on viability and forward/side scatter parameters consistent with single leukocytes. Non-B-cell populations, including T cells, macrophages, and other lymphocytes (CD3+, CD8+, CD14+), were excluded, and positive gating was applied to identify CD20+, CD27+, IgG+, and POWVI or POWVII EDIII+.

Isolated B cells that met these criteria (~0.1% of PBMCs; Fig. S1) were sorted into individual PCR tubes containing lysis buffer. Following lysis, cDNA was synthesized using random hexamer primers. Variable domains of immunoglobulin heavy and light chains, V_H_ and V_L_, were then amplified by a nested PCR strategy (41). Gibson assembly was used to clone into pMAZ-IgH/IgL vectors, which were then transfected in ExpiCHO cells to express human IgG1 mAbs (42). From the single-cell sorting using POWVI EDIII as bait, 136 B cells were isolated, from which we recovered 42 matching V_H_ and V_L_ sequences, resulting in the expression of 57 mAbs (Table S1). In some cases, multiple V_H_ or V_L_ domain sequences were detected from a single collection well, and, in those cases, all pairings were produced and tested. Similarly, using POWVII EDIII as bait, 144 B cells were isolated, and 17 expressed mAbs were recovered (Table S1). The binding properties of all 74 expressed mAbs to the respective POWVI/II EDIII antigen were evaluated by ELISA using 30 nM and 300 nM concentrations (Fig. 1C). Most POWVI and POWVII mAbs exhibited antigen-specific binding to their respective EDIII proteins; no binding was observed against BSA-coated wells. After evaluating antibodies for their relative binding signal above background controls as well as their binding consistency across concentrations, 23 POWVI mAbs and 5 POWVII mAbs were identified for further characterization. While binding for both groups of mAbs was modest, it was specific, as none of the mAbs showed any significant binding to control wells coated with 1% BSA.

We sought to broaden our panel beyond EDIII by performing additional B-cell isolation procedures using the POWV E ectodomain as the sorting bait. The yield from these single-cell sorts was lower; 18 B cells were isolated, resulting in seven expressed mAbs (Table S1). Among the seven POWV E-isolated mAbs, two mAbs, E.15H1L1 and E.PVE13, were prioritized based on their binding to POWV E and neutralizing activity (see below). E.15H1L1 and E.PVE13 exhibited stronger binding to E, with *K*_*D*_^app^ values of 8.55×10^−10^ M and 4.55×10^−9^ M, respectively. E.15H1L1 also reacted strongly to both POWVI and POWVII EDIII by ELISA, indicating that it engages an epitope of EDIII (Fig. S2). In contrast, E.PVE13 did not bind to POWV EDIII, indicating that this antibody recognizes an epitope elsewhere on the POWV E glycoprotein.

### Virus neutralization

To characterize the neutralizing potential of the mAbs, we focused our analysis on the 31 mAbs that bound to POWV antigens at or above the average ELISA signal of the population of mAbs. This panel included 23 POWVI EDIII-sorted mAbs, five POWVII EDIII-sorted mAbs, and three POWV E-sorted mAbs. All 31 mAbs were assessed at BSL2 in neutralization assays using POWV RVPs, with POWVI mAbs tested against POWVI RVPs, POWVII mAbs tested against POWVII RVPs, and POWV E mAbs tested against both POWVI and POWVII RVP strains (Fig. S3). The previously described chimeric antibody m158.25, which has murine variable domains and human constant domains, was used as a positive control (36). A range of neutralizing activities was observed with several (including E.15H1L1 and E.PVE13) exhibiting high levels of neutralization at both antibody concentrations.

To further evaluate the potency of the top nine neutralizing mAbs, we conducted studies with authentic POWV under BSL3 conditions using a focus reduction neutralization test (FRNT). Differences in apparent potency between RVP-based neutralization assays and authentic virus FRNT assays have been described previously (7). RVP assays can yield lower IC_50_ values due to differences in particle maturation state, epitope accessibility, and assay sensitivity, although relative neutralization trends are generally preserved. In this study, both assays were performed. The RVP assay measures single-round infection using a sensitive GFP reporter readout, whereas FRNT assays require sustained suppression of infectious virus over multiple days. In our previous work with chimeric murine POWV mAbs, we found that neutralization assays against POWV RVPs (BSL2 conditions) and against the POWVII MA51240 strain (BSL3 conditions) were the most predictive of *in vivo* protective efficacy (36). The MA51240 POWV strain was isolated from *I. scapularis* ticks in Spooner, Wisconsin, in 2008 (37, 43). It carries a K10N mutation in the M protein, relative to other isolates, which alters the presentation of E protein epitopes and increases sensitivity to antibody-mediated neutralization (37).

**Fig 2:**
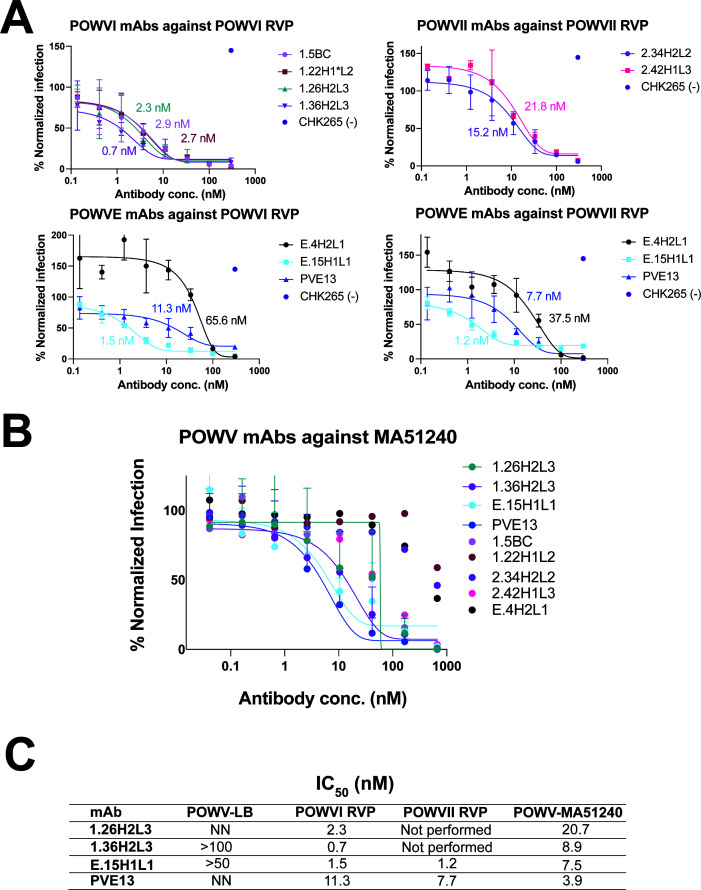
Neutralization profiles of anti-POWV mAbs. (**A**) Neutralization of POWVI and POWVII RVPs by POWVI, POWVII, and POWV E mAbs. Representative data from two independent experiments, each performed in triplicate, are shown for each subset of antibodies (POWVI-isolated, POWVII-isolated, and POWV E-isolated) against their respective antigens. CHK265 is included at a high concentration (300 nM) as a negative control. (**B**) Neutralization of POWV MA51240 by the nine most potent human mAbs. Representative data from two independent experiments, performed in duplicate, are shown. (**C**) Table of representative IC_50_ values for the experimental plots shown in panel A. The IC_50_ values for the four most potent human mAbs against authentic POWV strains (POWV LB and POWV MA51240) and POWVI and POWVII RVPs are shown. NN and ND mean non-neutralizing and not done, respectively.

Full dose-response neutralization curves against MA51240 were performed for these top nine neutralizing mAbs, which demonstrated an IC_50_ range of 3.9 to ~840 nM (Fig. 2B). The top four neutralizing mAbs, POWVI mAbs 1.26H2L3 and 1.36H2L3 and POWV E mAbs E.15H1L1 and E.PVE13, exhibited IC_50_ values ranging from 3.9 to 21 nM (Fig. 2C). When tested against the POWV LB strain, however, these mAbs demonstrated limited or no neutralization, with 1.26H2L3 and E.PVE13 showing no measurable activity, E.15H1L1 exhibiting an IC_50_ > 50 nM, and 1.36H2L3 showing an IC_50_ > 100 nM (Fig. 2C). These results indicate there is a significant divergence in susceptibility to neutralization between POWV LB and POWV MA51240. Notably, previously characterized murine mAbs m158.25 and m158.36 neutralize MA51240 but not LB *in vitro*, with m158.25 still conferring protection against POWVII-SPO *in vivo* (36).

To investigate the potential for cross-reactivity with other orthoflaviviruses, the top four neutralizing mAbs were evaluated for binding and neutralization of WNV (Fig. S4), a distantly related, mosquito-borne orthoflavivirus that shares 37.6% amino acid identity with POWVII in the prM-E region (44). ELISA analysis revealed that all four mAbs bound to WNV E, with E.PVE13 and 1.36H2L3 being the most cross-reactive. E.PVE13 exhibited the strongest cross-reactivity based on optical density signals at 300 nM and 30 nM. Unexpectedly, 1.36H2L3 bound more strongly to WNV E than to POWVI EDIII, the antigen used to identify this mAb, and thus exhibits cross-reactivity between the two viruses. It should be noted that the WNV E antigen used was produced recombinantly in *Escherichia coli* and refolded from inclusion bodies and therefore lacks native glycosylation, which may affect epitope presentation and antibody binding. None of the four mAbs exhibited significant neutralizing capabilities against authentic WNV in FRNTs, indicating that cross-reactive binding does not necessarily translate to functional neutralization.

### Binding profiles and epitope binning

**Fig 3:**
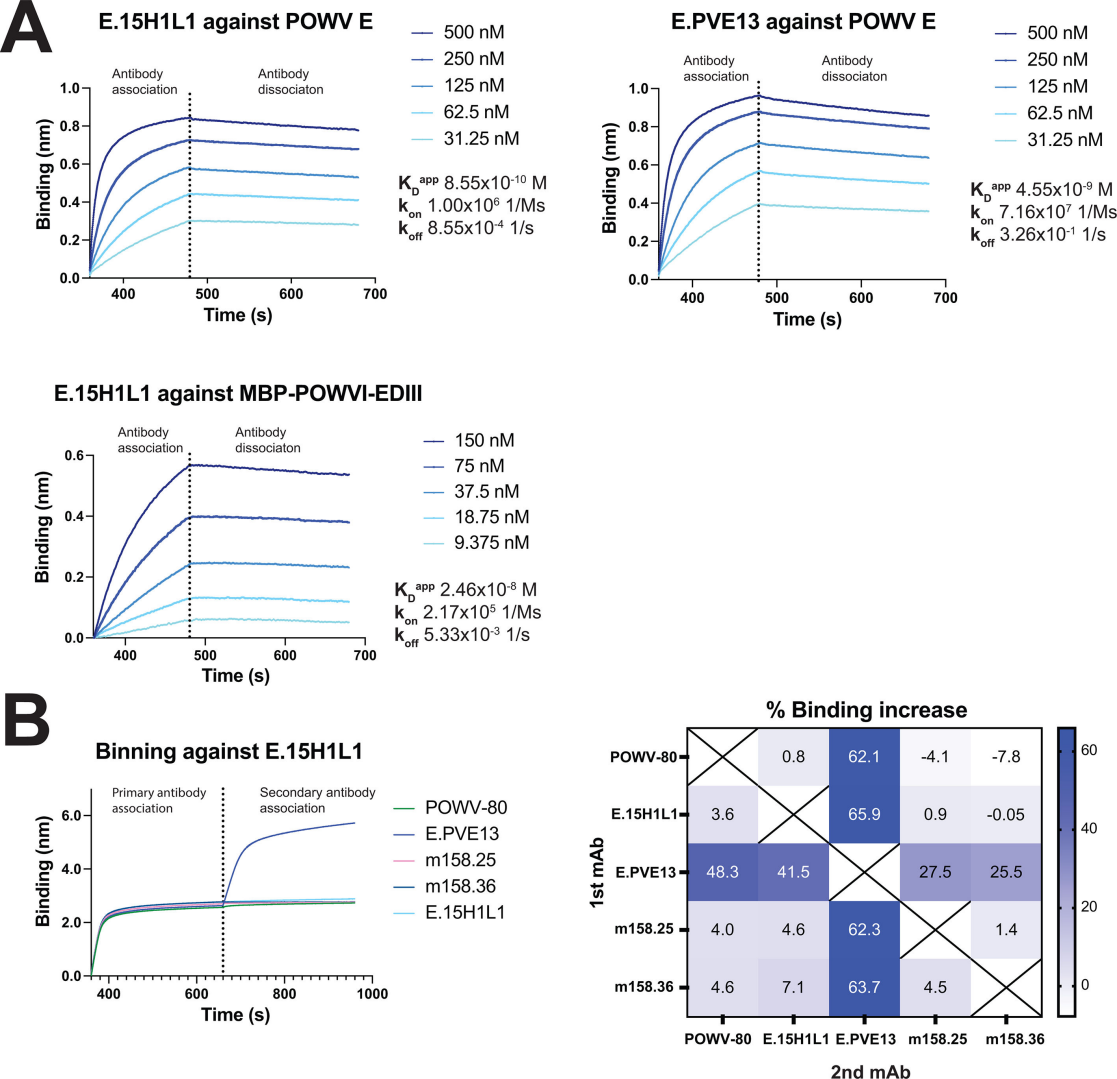
Binding profiles and epitope binning of anti-POWV mAbs. (**A**) Binding of neutralizing anti-POWV E mAbs to POWVI E or MBP-POWVI-EDIII measured by BLI. A representative data set from two independent experiments is shown. (**B**) Two-phase epitope binning of neutralizing mAbs against POWVI E by BLI. POWVI E was immobilized on the sensor and first bound by mAb E.15H1L1, followed by the addition of the indicated second mAb. Additional pairwise competition experiments were performed with POWV-80, E.15H1L1, E.PVE13, m158.26, and m158.35 in both orientations. The heatmap indicates the percent binding increase following addition of the indicated second mAb. Representative data from two independent experiments are shown.

The binding affinities of the top four neutralizing mAbs (1.26H2L3, 1.36H2L3, E.15H1L1, and E.PVE13) were evaluated using biolayer interferometry (BLI). Anti-human Fc sensors were used to immobilize the antibodies, which were then exposed to different concentrations of antigen to assess binding kinetics. For characterization of POWVI EDIII-isolated mAbs 1.26H2L3 and 1.36H2L3, we used a maltose binding protein fusion to EDIII (MBP-POWVI-EDIII) as the analyte. This approach was based on prior observations that fusion of orthoflavivirus EDIII to MBP improves detection of binding by EDIII mAbs via BLI, as the binding signal is proportional to the molecular weight of the binding analyte (36). Both mAbs demonstrated moderate binding to POWVI EDIII via ELISA (OD_450_ range ~0.5–0.75 at 300 nM), but no detectable binding activity to the MBP-POWVI-EDIII via BLI despite the above observation that the mAbs exhibited neutralizing activity (Fig. S5) (45–47). We further examined binding of E.15H1L1 and E.PVE13 to POWV E (Fig. 3A). Both mAbs exhibited strong binding to POWV E with *K_D_*^app^ values of 0.85 nM and 4.55 nM, respectively. Additionally, E.15H1L1 bound strongly to MBP-POWVI-EDIII, supporting the hypothesis that most or all of its epitope lies within the EDIII domain.

Recent work characterizing murine POWV mAbs has provided insight into the epitopes targeted by neutralizing antibodies. The protective murine antibody POWV-80 binds to the C-C' loop of EDIII (37). Additionally, two neutralizing murine mAbs m158.25 and m158.36 compete with POWV-80 via BLI epitope binning, suggesting that these mAbs also engage this region of EDIII (36). To assess whether the neutralizing human mAbs recognize a similar epitope, two-phase epitope binning was conducted via BLI. In this experiment, biotinylated POWV E protein was loaded onto streptavidin sensors, followed by primary and then secondary antibodies. A binding signal during the secondary antibody association phase indicates that the ternary complex can be formed, whereas lack of binding indicates that the primary and secondary antibodies compete for the same epitope. As an example, two-phase binding of E.15H1L1 followed by POWV-80, E.PVE13, m158.25, or m158.26 is shown in Fig. 3B. The epitope binning analysis was expanded in this format with all mAbs in the panel against one another and in both orientations of binding to generate the heatmap in Fig. 3B.

Consistent with previous work, m158.25 and m158.36 failed to bind to POWV E when POWV-80 was pre-bound. Similarly, E.15H1L1 showed no binding, indicating that m158.25, m158.36, and E.15H1L1 all engage a similar or proximal epitope as POWV-80. In comparison, E.PVE13 bound the POWV-80/POWV E complex, indicating that it engages a spatially distinct epitope from POWV-80. These interactions are summarized in the heatmap, which depicts the percent binding increase following the addition of the second antibody (Fig. 3B). This is consistent with the ELISA data showing no binding of E.PVE13 to POWV EDIII.

### Sequence analysis of human mAbs

Sequence analysis showed that all four top neutralizing mAbs derive from common immunoglobulin germline genes (Tables S2 and S3). 1.26H2L3, 1.36H2L3, and E.PVE13 all originate from IGHV3, whereas E.15H1L1 is encoded by IGHV5. For the light chains, E.PVE13 and 1.36H2L3 are derived from IGKV1, whereas E.15H1L1 and 1.26H2L3 both originate from IGKV3. The heavy chain CDR3 regions spanned 12–20 amino acids, consistent with the range reported for other orthoflavivirus-specific antibodies (18, 48).

### Protective capacity of mAbs in mice

**Fig 4:**
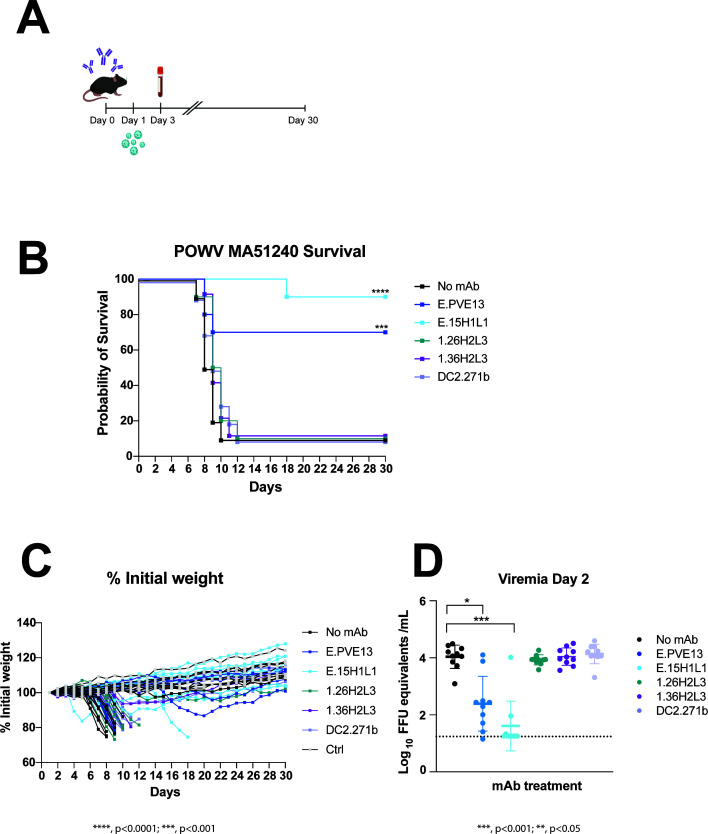
Human mAbs confer protection against POWV in lethal challenge model. (**A**) Scheme of challenge. (**B–D**) Six-week-old male C57BL/6J mice were treated with human mAbs (250 μg/mouse) administered via intraperitoneal injection in a 100-µL total volume. The following day, mice were inoculated with 10^2^ focus-forming units of POW-MA51240 subcutaneously in the footpad. Survival (**B**), weight loss (**C**), and viremia (**D**) were monitored, with the dotted line in panel D representing the limit of detection. Results are pooled from two independent experiments (*n* = 10). Survival was analyzed using a log-rank (Mantel-Cox) test comparing each group to the MA51240-only control. Weight-loss curves were plotted as mean ± SEM through day 9 due to survival bias; area under the curve (AUC) was computed and analyzed using Brown-Forsythe and Welch analysis of variance (ANOVA) with Dunnett’s T3 multiple-comparison test, comparing each group to the control. Viral loads were analyzed using a Kruskal-Wallis test with Dunn’s post-hoc test, and the median of each group is indicated with a line.

Based on their ability to neutralize the authentic POWVII MA51240 strain *in vitro*, four human mAbs (1.26H2L3, 1.36H2L3, E.15H1L1, and E.PVE13) were tested for their protective ability in mice challenged with MA51240 (Table S4). Six-week-old male C57BL/6J mice were treated with the POWV mAbs (250 µg/mouse) one day prior to virus infection and then monitored for survival for 4 weeks (Fig. 4A).

Nearly all (90%) of mice not receiving antibody treatment and 90% of mice treated with the negative-control antibody (DC2.271b, anti-chikungunya virus) succumbed to infection by days 10 and 12, respectively (Fig. 4B). Treatment with 1.26H2L3 or 1.36H2L3 resulted in similar results: 90% of the mice succumbed to infection by day 11 (1.36H2L3) or day 12 (1.26H2L3). In contrast, 90% of mice given E.15H1L1 and 70% of those given E.PVE13 survived the challenge. Body weight, expressed as a percentage of initial weight, was consistent with survival outcomes (Fig. 4C).

Viral RNA levels in the serum were quantified by qRT-PCR to evaluate the effect of antibody treatment on infection (Fig. 4D). Consistent with survival rates and weight-retention data, untreated mice, as well as mice treated with 1.26H2L3, 1.36H2L3, or the negative-control antibody DC2.271b, exhibited comparable viral loads in serum of approximately 4 log_10_ focus-forming units (FFU) equivalents per mL. In contrast, mice treated with E.PVE13 and E.15H1L1 showed reduced viral RNA levels, with E.15H1L1-treated mice displaying the lowest levels (mean: 1.6 log_10_ FFU equivalents/mL) and E.PVE13-treated mice maintaining slightly higher but still significantly reduced loads (mean: 2.4 log_10_ FFU equivalents per mL).

The *in vivo* protection profiles correlated with the neutralization potencies measured *in vitro*. Antibodies E.15H1L1 and E.PVE13, which demonstrated the strongest neutralizing activity against the MA51240 strain, conferred the greatest protection in mice, whereas the less potent neutralizers 1.26H2L3 and 1.36H2L3 offered limited protection.

## DISCUSSION

Here, we isolated human mAbs targeting POWV EDIII and the full E ectodomain from a convalescent patient using single B-cell sorting. We initially focused on isolating EDIII-isolated antibodies, given that this domain often contains potent neutralizing epitopes in other orthoflaviviruses. However, the limited binding activity observed among these mAbs prompted us to expand our strategy to include sorting with the full-length E ectodomain. We ultimately developed a panel of 81 human POWV mAbs: 57 POWVI EDIII-isolated, 17 POWVII EDIII-isolated, and seven E-isolated mAbs. These antibodies exhibited a range of binding and neutralization abilities. We identified two human mAbs (E.15H1L1 and E.PVE13) that conferred protection *in vivo*.

Both POWVI and POWVII EDIII-isolated mAbs exhibited low-to-moderate binding to their respective antigens via ELISA, but some were able to neutralize the virus efficiently *in vitro*. This observation suggests that recognition of some conformational epitopes may extend outside EDIII, such as EDI and EDII, or span multiple domains or quaternary epitopes formed across E dimers, as has been described previously for DENV and ZIKV (49, 50). For other orthoflaviviruses, EDIII-directed antibodies typically comprise a minor fraction of the human antibody repertoire but can be potently neutralizing, whereas the most potent human antibodies recognize quaternary epitopes spanning the E dimer (18, 51, 52).

To learn more about which epitopes were engaged by the mAbs, we performed competition assays using POWV-80, a cross-neutralizing murine mAb known to target the C-C' loop of EDIII (37). Three of the neutralizing mAbs (E.15H1L1, m158.25, and m158.36) competed with POWV-80, indicating that they likely target proximal epitopes on EDIII. In contrast, E.PVE13 did not compete with POWV-80, suggesting that it targets a distinct site, likely outside of EDIII. E.PVE13, which was isolated using the full E ectodomain, still neutralizes and confers protection *in vivo*, highlighting the presence of multiple distinct protective epitopes on E.

To evaluate neutralization potency, we performed FRNTs using the lineage II MA51240 strain, which more accurately predicted *in vivo* protective activity of murine anti-POWV mAbs compared to neutralization assays with the lineage I LB or the lineage II Spooner strains (36). Compared to these older strains, MA51240 contains a K10N substitution in the M protein, a mutation associated with enhanced sensitivity to antibody neutralization, possibly due to its effects on virus maturation (37). The EDIII antigens used in B-cell sorting were designed based on the LB and Spooner strains, with matching sequences used for RVP production. However, some EDIII-isolated mAbs that neutralized POWVI RVPs failed to neutralize the authentic LB virus, suggesting that differences in viral maturation state between authentic virus and RVPs may influence epitope accessibility and neutralization sensitivity (37).

While previous studies have shown protective effects of murine mAbs against POWV (36, 37), fully human mAb-based immunotherapies offer clinical advantages, including reduced risk of adverse reactions such as serum sickness or anaphylaxis. In this study, antibodies were evaluated at a single prophylactic dose (250 μg/mouse), and dose-response or therapeutic (post-exposure) studies were not performed. Future work will be required to determine the minimal protective dose and evaluate therapeutic efficacy following established infection. In addition, viral burden was measured only in serum at day two post-infection, which provides limited insight into central nervous system (CNS) infection. We did not directly assess viral dissemination to the CNS or monitor neurological disease manifestations in this study and therefore cannot determine the precise cause of death in untreated animals. Given the neurotropic nature of POWV, future studies evaluating viral dissemination to the CNS and its relationship to antibody-mediated protection are critical for further defining the protective mechanisms of these mAbs. It is also important to note that *in vivo* protection studies described here were performed using the lineage II MA51240 strain, and therefore the protective efficacy of these antibodies against other POWV strains or lineages remains to be determined. Although E.15H1L1 and E.PVE13 neutralized both POWVI and POWVII RVPs *in vitro*, they showed limited or no neutralization against the authentic lineage I LB strain. Therefore, additional studies will be required to determine whether these antibodies confer protection across additional POWV strains and lineages. Despite these limitations, the human mAbs E.15H1L1 and E.PVE13 described here represent candidates for further preclinical therapeutic development.

## MATERIALS AND METHODS

### Cell lines, viruses, and POWV E protein

Following the manufacturer’s instructions, ExpiCHO-S cells (Gibco) were kept in ExpiCHO expression media and maintained at 37°C and 5% CO_2_. Vero cells (CCL-81, ATCC) were maintained in Dulbecco’s modified Eagle medium (DMEM; Invitrogen) containing either 5% or 10% fetal bovine serum (FBS; Omega Scientific) and 100 U/mL penicillin-streptomycin (P/S; Invitrogen) at 37°C in the presence of 5% CO_2_. POWV lineage I strain LB was obtained from World Reference Center for Emerging Viruses and Arboviruses (K. Plante and S. Weaver, University of Texas Medical Branch, Galveston, TX). POWVII strain MA51240 was isolated from ticks collected in Wisconsin (43). All viral stocks were propagated on Vero cells at 37°C for 72 h. Viral titer was determined by focus-forming assay (FFA). The POWV E protein was provided by Zachary Bornholdt and Crystal Moyer of Mapp Biopharmaceutical.

### Expression and purification of POWVI/II EDIII

Genes encoding POWVI and POWVII EDIII (GenBank accession numbers NC_003687 and HM440558) were codon optimized for *E. coli* expression, synthesized (IDT), and cloned into the pET His_6_MBP vector (Addgene plasmid #29656) using Gibson assembly (NEB) (36). Resulting constructs were transformed into BL21(DE3) cells for recombinant protein expression (New England Biolabs).

Overnight starter cultures in 2×YT supplemented with kanamycin were used to inoculate expression cultures, which were grown at 37°C and 220 rpm. Protein expression was induced at an OD_600_ of 0.6–0.8 with 0.4 mM IPTG for 18 h at 22°C with shaking at 220 rpm. Centrifugation at 4,000 g for 20 min at 4°C was used to pellet the bacteria. Bacterial pellets were resuspended in lysis buffer (50 mM Tris pH 8.0, 250 mM NaCl) and lysed by sonication. Insoluble material was removed by centrifugation at 17,000 g for 30 min at 4°C, and clarified lysates were incubated with Ni-NTA resin (GoldBio) for 2 h at 4°C. The resin was washed sequentially with wash buffer 1 (50 mM Tris-HCL pH 8.0, 250 mM NaCl, 10 mM imidazole) and then wash buffer 2 (50 mM Tris-HCl pH 8.0, 250 mM NaCl, 30 mM imidazole). Elution buffer (50 mM Tris-HCl pH 8.0, 250 mM NaCl, 250 mM Imidazole) was then used to elute the bound EDIII-MBP fusion proteins.

Eluted EDIII-MBP fusion proteins were dialyzed in buffer 1 (50 mM Tris-HCl pH 8.0, 250 mM NaCl) for 4 h at 4°C. To isolate the POWV EDIII, cleavage with Tobacco Etch Virus protease was performed overnight (1:50 w/w). Cleavage products were then dialyzed in low salt buffer 2 (50 mM Tris pH 9.0, 20 mM NaCl) for at least 4 h at 4°C. The cleaved EDIII and MBP proteins were then separated via anion exchange chromatography using MonoQ 10/100 GL, as previously described (36). Proteins were eluted with a linear NaCl gradient buffer of 50 mM Tris-HCl pH 9.0, 1 M NaCl. SDS-PAGE was used to confirm fractions containing EDIII protein, which were then pooled and buffer exchanged into storage buffer (50 mM Tris-HCl pH 8.0, 250 mM NaCl).

### WNV E protein

The expression vector of WNV New York 1999 E protein (GenBank accession number ABA62343.1, MW: 43.5 kDa) was cloned by restriction digestion and ligation of the codon-optimized E protein sequence (AA: 291–694) into a pET21 vector (Novagen) between the BamHI and XhoI sites (53). Following sequence confirmation by Sanger sequencing (Azenta Genewiz), plasmids were transformed into BL21(DE3) (New England Biolabs). Bacteria were grown in Luria broth, induced with 0.5 mM isopropyl thiogalactoside (IPTG), and pelleted. Following induction, bacteria were lysed by sonication in the presence of lysozyme. The E protein was recovered as insoluble aggregates within the inclusion bodies. The E protein aggregates were denatured in the presence of guanidine hydrochloride and β-mercaptoethanol and refolded by slowly diluting out the denaturing reagents in the presence of L-arginine, EDTA, PMSF, reduced glutathione, and oxidized glutathione. Refolded E protein was purified by size-exclusion chromatography on a S200 Increase 10/300 column (Cytiva) in 20 mM HEPES pH 8.0, 150 mM NaCl, and fractions corresponding to the expected molecular weight were collected.

### Production of RVPs

POWV lineage I and II RVPs were produced as previously described (36). Codon-optimized plasmids encoding the structural proteins C, prM, and E of POWVI (GenBank accession number NC_003687.1) and POWVII (GenBank accession number HM440558.1) were synthesized in a pCAGGS vector (Epoch Life Science). These plasmids were then co-transfected in 293FT cells (Thermo Fisher) with a WNV subgenomic replicon plasmid, pcDNA6.2-WNIIrep-GFP/zeo (a gift from Dr. Ted Pierson, NIH). Transfections were performed using 3 µg of POWV C-prM-E and 9 µg of WNV replicon DNA (1:3 ratio) per 15 cm plate. Cells were cultured in high glucose DMEM (Gibco) supplemented with 10% heat-inactivated FBS (Atlanta Biologicals), 1% penicillin-streptomycin, and 1% GlutaMAX. After 8 h, media was replaced with low glucose DMEM containing 5% heat-inactivated FBS and 25 mM HEPES pH 7.3. Supernatants containing RVPs were harvested after 72–96 h at 37°C and 5% CO_2_, clarified by centrifugation (4,000 rpm, 4°C, 15 min, 2×), and pelleted by ultracentrifugation for 4 h at 28,000 rpm through a 2 mL 30% sucrose cushion (SW28 rotor, Optima LE-80K, Beckman Coulter). Pellets were resuspended overnight on ice in PBS (pH 7.4), aliquoted, and stored at −80°C.

### Isolation of PBMCs

PBMCs were isolated from donor plasma using density gradient centrifugation. Plasma was diluted 1:1 with Hanks’ Balanced Salt Solution (HBSS), layered over 15 mL of Histopaque in a Leucosep tube, and centrifuged at 800 × g for 1 min at room temperature. The PBMC layer was collected, diluted to 50 mL with HBSS, and centrifuged at 450 × g for 10 min. The resulting pellets were resuspended in 50 mL of HBSS and centrifuged again under the same conditions. The final cell pellet was resuspended in freezing medium (90% FBS, 10% DMSO) at a concentration of 2 × 10^7^ PBMCs/mL and stored in liquid nitrogen.

### Isolation of POWV mAbs by single B-cell sorting

Human mAb isolation was performed as previously described (36, 54, 55). Antigen-reactive B cells were isolated from patient PBMCs by FACS. PBMCs were stained on ice with the following fluorophore-conjugated human-specific antibodies: anti-CD3 (PE/Cy7), anti-CD8 (PE/Cy7), anti-CD14 (PE/Cy7), anti-CD20 (Pacific Blue), anti-CD27 (APC), and anti-IgG (FITC) (Thermo Fisher Scientific). Antibodies were used at a 1:50 dilution, except anti-IgG-FITC, which was 1:25. The PBMCs were incubated with biotinylated antigen (POWVI EDIII, POWVII EDIII, or POWVI E), prepared using EZ-Link NHS-PEG4-Biotin (Life Technologies), and then complexed with streptavidin-PE. The POWVI and POWVII EDIII sorting antigens used for antibody selection were expressed and purified in a folded, native-like conformation (36). Cells with T-cell or macrophage markers were excluded. IgG+ and antigen+ cells were single-sorted into PCR strips containing lysis buffer and frozen on dry ice before synthesizing cDNA by RT-PCR. Nested PCR was used to amplify the IgH and IgK variable domains, which were then cloned into the mammalian pMAZ vector via Gibson assembly for sequencing and recombinant expression (42). Using the ExpiCHO Expression System (Thermo Fisher Scientific), the resulting plasmids were then transiently co-transfected in ExpiCHO cells and purified by protein A chromatography.

### ELISA with POWV E and EDIII

ELISA assays were performed to evaluate binding of the mAbs to their respective antigens. POWVI EDIII, POWVII EDIII, and POWVI E ectodomain were coated onto 96-well half-area high binding plates (Costar) at 200 ng/well overnight at 4°C. Wells were blocked with 1% BSA at 28°C for 2 h and washed five times with PBS-T (PBS pH 7.4, 0.05% Tween-20). The isolated mAbs were then added at three different concentrations (3, 30, and 300 nM) and incubated at 28°C for 1 h. 1% BSA and a non-flavivirus mAb CHK265 were used as negative controls. After plates were washed, protein A-horseradish peroxidase (HRP) (Life Technologies) was added to the wells at a 1:2,000 dilution and incubated at 28°C for 1 h. Plates were washed, developed using 3,3’5,5’-tetramethylbenzidine (TMB), quenched with sulfuric acid, and read at an absorbance of 450 nm on a Synergy H4 Hybrid reader (BioTek).

### Binding kinetics by BLI

Binding kinetics of the mAbs to MBP-tagged POWVI EDIII were performed as previously described by BLI on an OctetRed96 system (ForteBio) (36). Anti-human Fc capture biosensors were used to immobilize the mAbs before dipping them into wells containing MBP-POWVI-EDIII. Kinetic parameters, including the association rate constant (*k*_on_), the dissociation rate constant (*k*_off_), and the apparent equilibrium dissociation constant (*K_D_*^app^), were then estimated using global data fitting to a 1:1 binding model. All analyses were performed using ForteBio Data Analysis Software version 10. Two-phase epitope binning was also performed via BLI by first immobilizing biotinylated POWVI E onto streptavidin-coated biosensors. After saturation with the primary mAb, sensors were then transferred into wells containing an equimolar mix (50 nM) of both the primary mAb and secondary mAb to assess competitive binding.

### RVP neutralization assay

Vero cells were seeded in a monolayer in a 96-well plate (Costar) in DMEM high glucose medium (Gibco) supplemented with 2% FBS (heat-inactivated, Atlanta Biologicals), 1% P/S (Gibco), 1% Glutamax (Gibco), and 25 mM HEPES (Gibco) at 18,000 cells/well. After a 1-day incubation, mAbs were serially diluted with a starting concentration of 300 nM in the supplemented DMEM high glucose media and incubated with the RVPs for 1 h. An unrelated mAb (CHK-265) was included at 300 nM as a negative control. The RVP-mAb mixture was then added in triplicate to the Vero cells and incubated at 37°C and 5% CO_2_ for 2 days. After 48 h, cells were fixed with 8% paraformaldehyde (Sigma) for 10 m and washed three times with PBS. Cell nuclei were then dyed with Hoechst-33342 (Life Technologies) in a 1:2000 dilution in PBS to provide the total cell count. Using a Cytation-5 automated fluorescence microscope (BioTek), viral infectivity was measured by automated enumeration of GFP+ cells versus total cell count. Nonlinear regression analysis with GraphPad Prism software was used to determine the half-maximal inhibitory concentration (IC_50_) of the tested mAbs.

### Authentic POWV and WNV neutralization assays

FRNTs were performed as described previously for other orthoflaviviruses (56). Serial dilutions of antibodies were incubated with 10^2^ FFU of POWV-MA51240, POWV-LB, or WNV for 1 h at 37°C. The previously published POWV-neutralizing mAb POWV-62 was included as a positive control to confirm assay performance (37). Antibody-virus complexes were added to Vero cells and incubated for another 1 h at 37°C. Finally, 1% (wt/vol) methylcellulose in MEM was added as overlay. After a 3-day incubation at 37°C, cells were fixed with 1% paraformaldehyde, permeabilized with 0.1% saponin, and stained for infection foci using 1 µg/mL of POWV-37 (37). Antibody-dose response curves were analyzed using nonlinear regression (GraphPad Software).

### *In vivo* challenge

C57BL/6J mice were purchased from The Jackson Laboratory and housed in a pathogen-free animal facility at Washington University in St. Louis. Male mice were used to minimize hormonal variability and improve statistical power, since this removes sex as a biological variable. In addition, previous work shows there is no difference in weight loss and survival among male and female mice infected with POWV (57). Virus inoculations were performed under anesthesia that was induced and maintained with isoflurane. Groups of 6-week-old male C57BL/6J mice were treated with mAbs (250 μg/mouse) diluted in PBS administered via intraperitoneal injection in a 100-µL total volume. This dose (~10–12 mg/kg) was selected based on previously published mAb protection studies in POWV and other orthoflavivirus mouse models using similar prophylactic dosing regimens (37, 58). The following day, mice were inoculated with 10^2^ FFU of POWV MA51240 subcutaneously in the footpad. Blood was collected from the animals by submandibular bleeds two days after virus inoculation. Mice were monitored daily for 30 days for weight loss and mortality and were euthanized if they lost ≥25% of their initial body weight or reached a moribund state.

### Quantification of viremia

Blood was collected from mice in SST BD Microtainer tubes at 2 days after infection and allowed to clot. After centrifugation, serum was collected, and RNA was extracted using the MagMAX Viral RNA Isolation Kit (Thermo Fisher Scientific). RNA was amplified using the TaqMan RNA-to-CT 1-Step kit (Cat no. 4,392,938, Thermo Fisher, USA) and PrimeTime qPCR Assay primers (IDT, USA) on QuantStudio 6 Flex System (Applied Biosystems, USA). Viral burden was quantified on a log₁₀ scale and expressed as viral RNA equivalents per milliliter of sample, based on a standard curve generated from serial 10-fold dilutions of viral RNA derived from known amounts of infectious virus. POWV primers (37) were 5′-GCAGCACCATAGGTAGAATGT-3′, 5′-CCACCCACTGAACCAAAGT-3′, and probe 5′-/56-FAM/TCTCAGTGG/Zen/TTG GAGAACACGCAT/3IABkFQ-3′.

## Data Availability

All data are available within the files associated with this paper.

## SUPPLEMENTAL MATERIAL

Supplemental figures and tables jvi.02105-25-s0001.pdf Fig. S1 to S5 and Tables S1 to S4.

ASM does not own the copyrights to Supplemental Material that may be linked to, or accessed through, an article. The authors have granted ASM a non-exclusive, world-wide license to publish the Supplemental Material files. Please contact the corresponding author directly for reuse.
