# Supplementary material for: Protective human antibodies against Powassan virus
Source: J Virol. 2026 Apr 30;100(5):e02105-25. doi: 10.1128/jvi.02105-25 (PMC13185606; doi:10.1128/jvi.02105-25)
Supplement: Supplemental figures and tables — Fig. S1 to S5 and Tables S1 to S4. [file jvi.02105-25-s0001.pdf]

Supporting Information for:

Protective human antibodies against Powassan virus

Georgia Fallon,[a] Stefanie P. Muraro,[b] Samuel Ailsworth,[a] Sean Hui,[c] Ryan J. Malonis,[a*] Alexandra L. Tse,[d] Emily Happy Miller,[d,f] Kartik Chandran,[d] Daved H. Fremont,[c,e] Michael S. Diamond,[b,c,e] Jonathan R. Lai[a#]

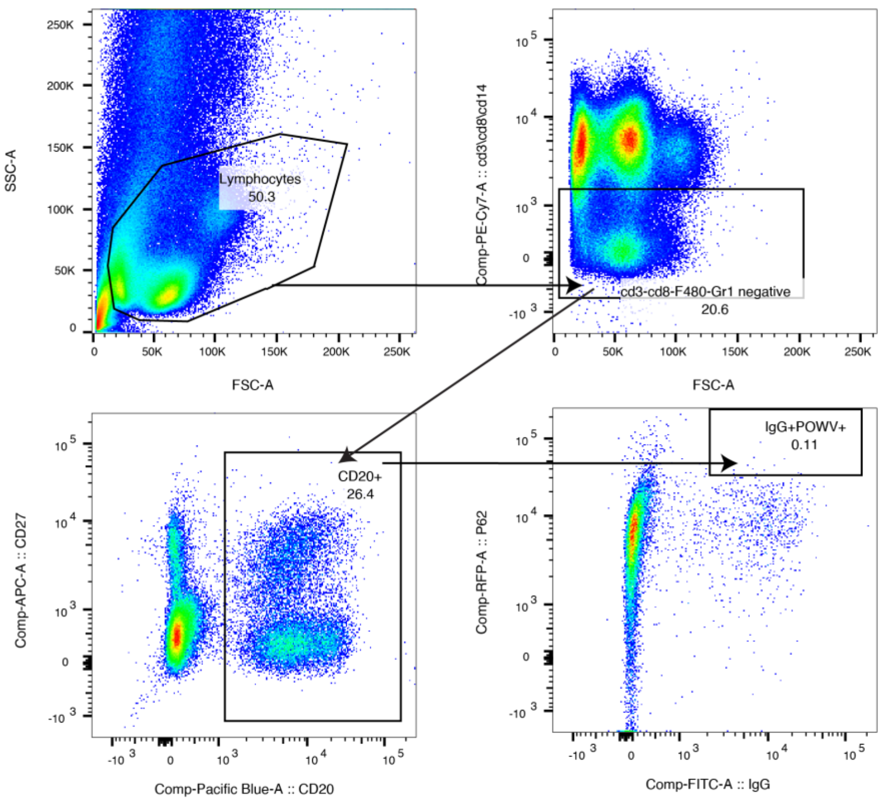

Figure S1. Gating strategy for the isolation of POWVI EDIII-reactive B cells from convalescent donor. Representative flow cytometric gating of human PBMCs is shown. Cells were gated for size and granularity. CD3+, CD8+, F4/80+, and Gr-1+ cells were excluded. CD20+, IgG+, and POWVI EDIII+ B cells were sorted into individual wells.

	POWVI EDIII	POWVII EDIII	POWV E
Isolated B cells	136	144	18
Matching HC & LC pairs	42	28	5
Expressed mAbs	57	17	7

Table S1. Total yield from mAb development pipeline. Yield for POWVI EDIII-, POWVII EDIII-, and POWV E-specific mAbs from isolated B cells to developed mAb.

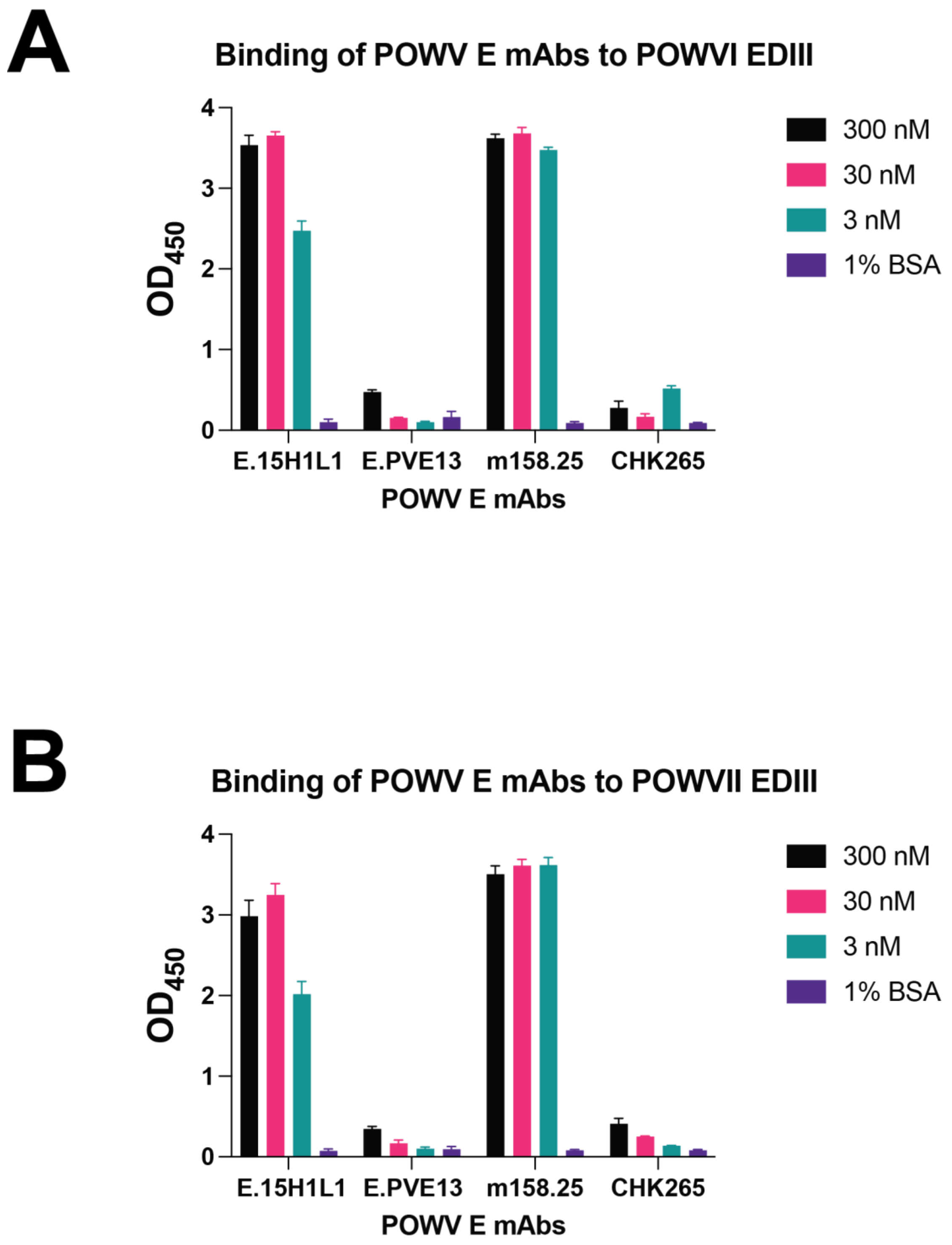

Figure S2. Binding of POWV E-isolated mAbs toward POWVI and POWVII EDIII. (**A**) Binding of POWV E-isolated mAbs against POWVI EDIII. (**B**) Binding of POWV E-isolated

mAbs against POWVII EDIII. Experiments performed twice independently in triplicate. EDIII-isolated murine mAb m158.25 is included as a positive control and unrelated mAb CHK265 is included as a negative control.

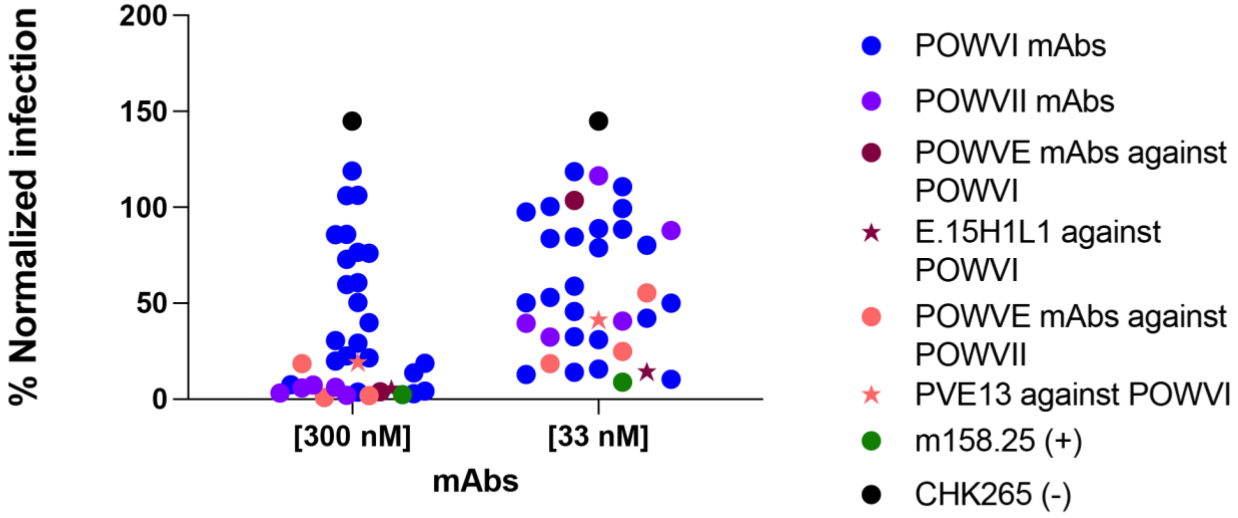

Figure S3. Neutralization profile of 31 anti-POWV mAbs against POWV RVP.
Plot of percent normalized infection for the 31 isolated mAbs at 300 nM and 33 nM. POWVI mAbs were tested against POWVI RVPs, POWVII mAbs were tested against POWVII RVPs, and POWV E mAbs were tested against both POWVI and POWVII RVP strains. Murine mAb m158.25 is used as positive control; Chikungunya virus-specific mAb CHK265 is used as negative control. Each data point represents the mean from two or more independent experiments done in triplicate.

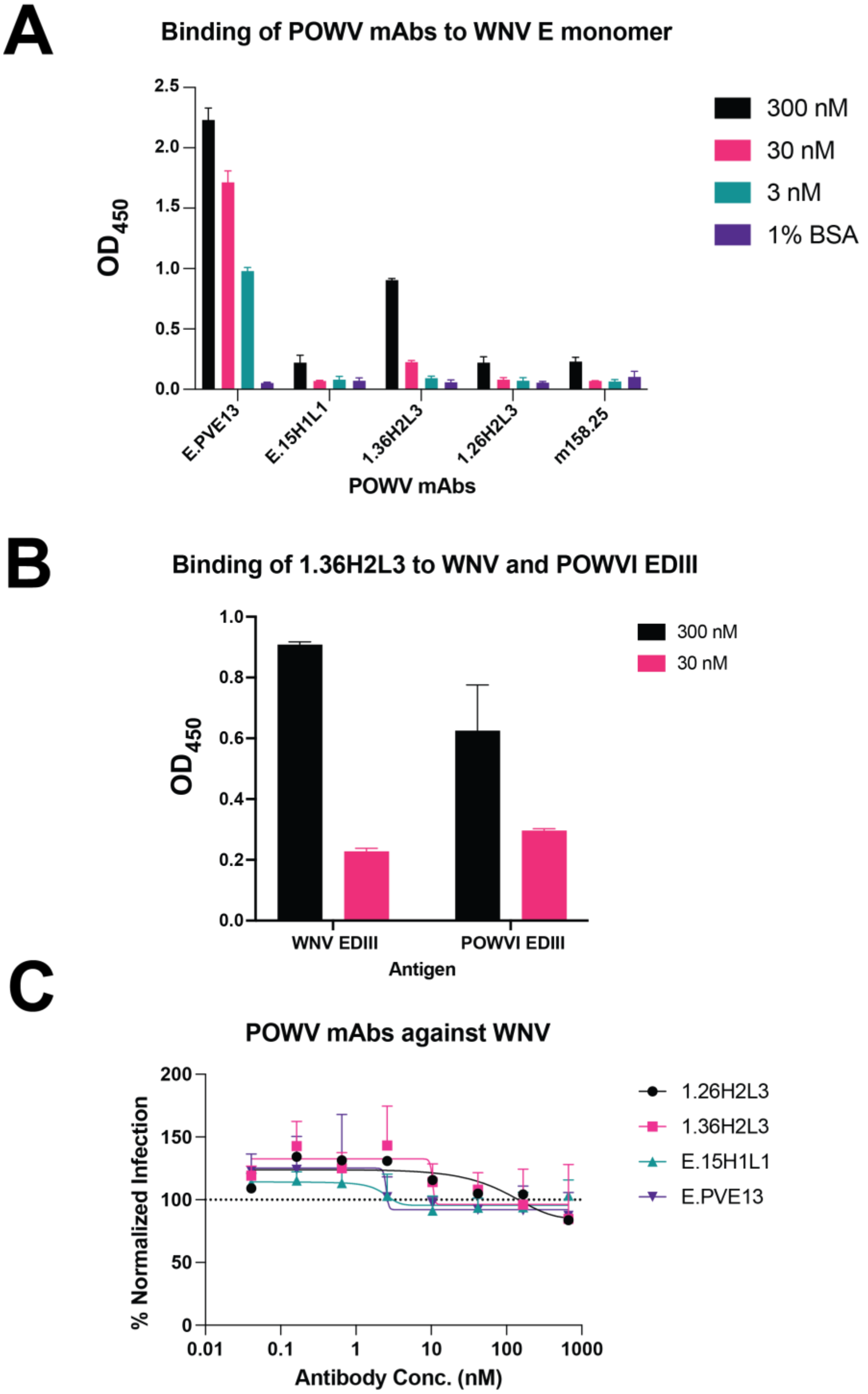

Figure S4. Binding and neutralization profile of POWV mAbs against WNV.
(**A**) Binding of POWV mAbs to WNV E. Experiment performed twice in triplicate. (**B**) Comparison of binding of POWVI-isolated mAb 1.36H2L3 toward WNV EDIII and POWVI EDIII. (**C**)Neutralization of WNV by POWV mAbs. Representative data from two independent experiments are shown.

A

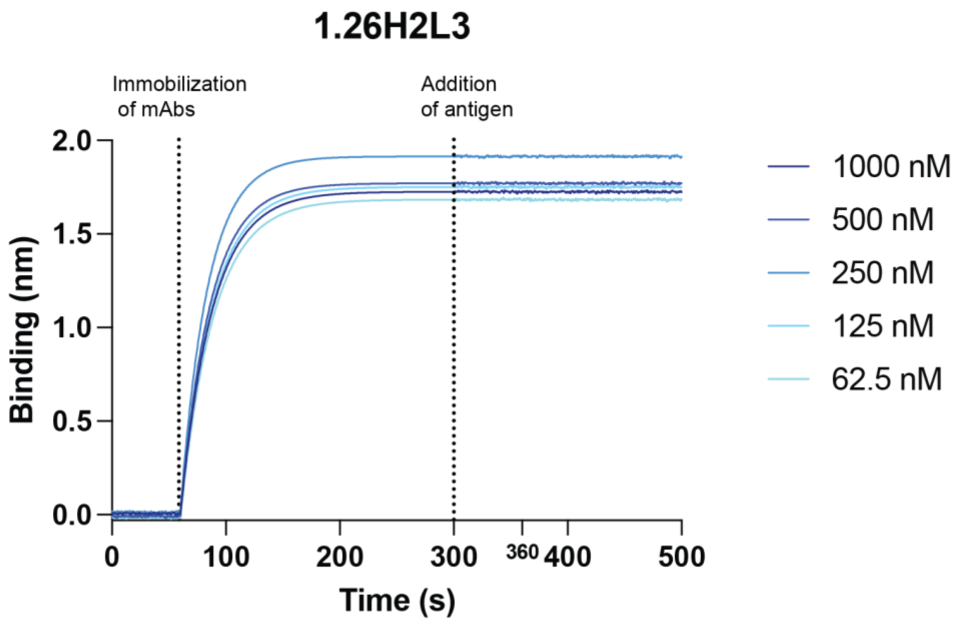

B

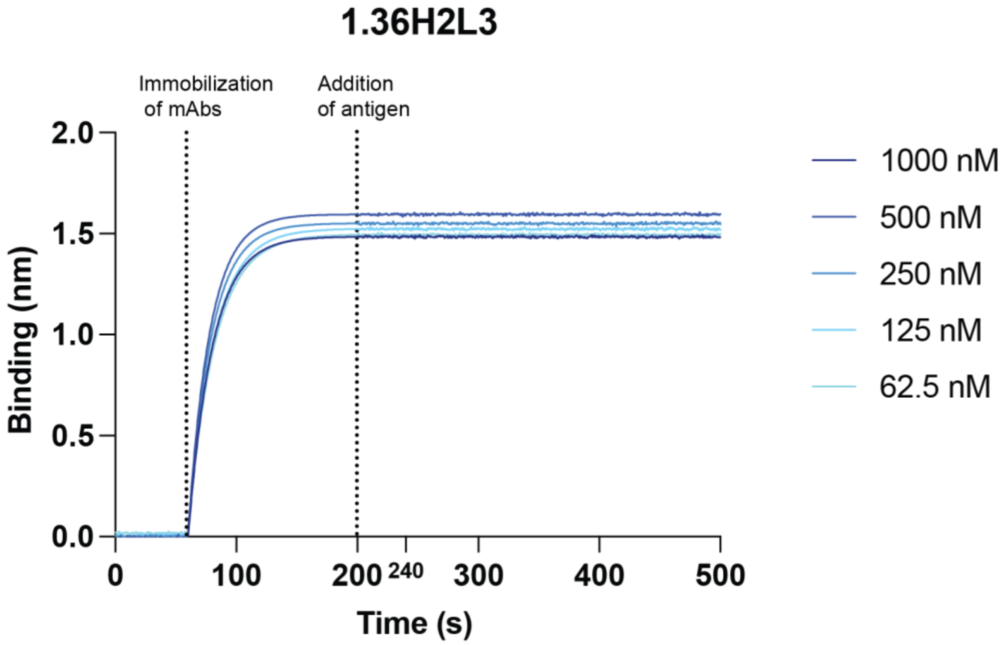

Figure S5. Binding profiles of 1.26H2L3 and 1.36H2L3 by BLI.
(**A**) Binding of 1.26H2L3 to MBP-POWVI-DIII by BLI. (**B**) Binding of 1.36H2L3 to MBP-POWVI-EDIII by BLI. The first dotted line represents the immobilization of the mAbs onto the sensors. The second dotted line represents the addition of antigen (MBP-POWVI-EDIII). Representative data from two independent experiments are shown.

mAb	V-gene	CDR lengths	CDR3
1.26H2L3	IGHV3	20	AIQGYCRSGRCPEGGDWFDP
	IGKV3	8	QQYGSSLT
1.36H2L3	IGHV3	13	VKDEDARGWRLDK
	IGKV1	9	LQDNSYPWT
E.15H1L1	IGHV5	14	ARIQYSSSWYDFDY
	IGKV3	11	QQGSNWPPALT
PVE13	IGHV3	12	TAFWSGDFYFDY
	IGKV1	6	QQSQRT

Table S2. POWVI and POWVE human mAb sequence profiles.

mAb	V_H sequence	V_L sequence
1.26H2L3	QVQLVQSGGGLVQPGGSLRL<br>SCAASGFSFRSFDMHWVRQT<br>TGKGLEWVSAIGTASDPYYL<br>ASVKGRFTISRENAKNSLYL<br>QIDSLRAEDTAVYYCARGAH<br>GGWGVAARPDWYFDLWGRGT<br>LVTVSS	DIVLTQSPGTLSLSPGERAT<br>FSCRASQSVSNSFLAWYQQK<br>PGQPPRLLIFGASSRATGIA<br>DRFSGSGSGTDFTLTISRLE<br>PEDFAVYYCQQYGSSLTFGG<br>GTKVDIK
1.36H2L3	QVQLVQSGGGVVQPGRSLRL<br>SCVASEFTFSSYGMHWVRQA<br>PGKGLEWVAVISYDGTEKYY<br>ADSVKGRFTISRDNSKYTLY<br>LQMNNLRAQGTGFYYCVKDE<br>DARGWRLDKWGQGTLVTVSS	DIVLTQTPSSLSASVGDRVT<br>ITCRASQDIRSDLGWFQQKP<br>GKVPKRLIYSASSLQTGVPS<br>RFSGSGSGTEFTLTISSLQP<br>EDFATYYCLQDNSYPWTFGQ<br>GTKVEIK
E.15H1L1	QVTLKESGPALVKPTQTLTL<br>TCTFSGFSLSTNGMCVGWIR<br>QPPGKALEWLALIDWDDNKY<br>YSTSLKTRLTISKDTSKNQV<br>VLRMTNVDPVDTATYYCARI<br>QYSSSWYDFDYWGQGTLVTVSS	EIVLTQSPDTLSLAPGERAT<br>LSCRASHSVSSYLAWYQQKP<br>GQAPRLLIYDASNRATGVPA<br>RFSGSGSGTDFTLTISSLEP<br>EDFAVYYCQQGSNWPPALTF<br>GGGTKVDIK
E.PVE13	QVQLVESGGGLVQPGRSLRL<br>SCAASGFTFSTYPMSWVRQA<br>PGKGLEWVSTISGSDGSTYY<br>ADSVKGRFTISRDSSKNTAY<br>LQMNSLRGEDTAVYYCTAFW<br>SGDFYFDYWGQGTLVTVSS	DIRLTQSPSSLSASVGDRVT<br>ITCRASQSIASYVNWYQQKP<br>GKAPKLLIYAASNLQNGVPS<br>RFSGRGSGTDFTLTISSLQP<br>EDFATYYCQQSQRTFGQGTK<br>VDIK

Table S3. POWVI and POWVE variable heavy and light chain sequences.

mAb	Selection bait	Binding capacity (OD_{450})	IC_{50} (POWVI RVP)	IC_{50} (POWVII RVP)	IC_{50} (POWV MA51240)	Cross-reactivity (WNV E)	Protective *in vivo*
1.26H2L3	POWVI EDIII	1.07	2.7 nM	ND	20.7 nM	No	No
1.36H2L3	POWVI EDIII	0.70	1.2 nM	ND	8.9 nM	Yes	No
E.15H1L1	POWV E	3.51	1.8 nM	1.5 nM	7.5 nM	No	Yes
E.PVE13	POWV E	2.96	13.6 nM	9.6 nM	3.9 nM	Yes	Yes

Table S4. Summary table for the top four mAbs evaluated *in vivo*. Table includes mAb name, the bait used to sort the mAb, the binding capacity (OD_{450}) of the mAb toward its sorting antigen at 300 nM, the representative IC_{50} value of the mAb under BSL2 conditions, the representative IC_{50} value of the mAb under BSL3 conditions, whether or not the mAb exhibited cross-reactivity toward WNV E, and the protective capacity of the mAb *in vivo*. ND = not done.
